# Pharmacokinetic and pharmacodynamic evaluation of nasal liposome and nanoparticle based rivastigmine formulations in acute and chronic models of Alzheimer’s disease

**DOI:** 10.1007/s00210-021-02096-0

**Published:** 2021-06-04

**Authors:** Sampath Kumar L. Rompicherla, Karthik Arumugam, Sree Lalitha Bojja, Nitesh Kumar, C. Mallikarjuna Rao

**Affiliations:** 1 Department of Pharmacology, Manipal College of Pharmaceutical Sciences, Manipal Academy of Higher Education, Manipal, Karnataka 576104 India; 2 Department of Pharmacology and Toxicology, National Institute of Pharmaceutical Education and Research, Hajipur, Bihar 844102 India

**Keywords:** PK-PD modeling, Amnesia, Neurodegeneration, Nanoformulation, Nose to brain delivery

## Abstract

With the increasing aging population and progressive nature of the disease, Alzheimer’s disease (AD) poses to be an oncoming epidemic with limited therapeutic strategies. It is characterized by memory loss, behavioral instability, impaired cognitive function, predominantly, cognitive inability manifested due to the accumulation of β-amyloid, with malfunctioned cholinergic system. Rivastigmine, a reversible dual cholinesterase inhibitor, is a more tolerable and widely used choice of drug for AD. However, rivastigmine being hydrophilic and undergoing the first-pass metabolism exhibits low CNS bioavailability. Nanoformulations including liposomes and PLGA nanoparticles can encapsulate hydrophilic drugs and deliver them efficiently to the brain. Besides, the nasal route is receiving considerable attention recently, due to its direct access to the brain. Therefore, the present study attempts to evaluate the pharmacokinetic and pharmacodynamic properties of nasal liposomal and PLGA nanoparticle formulations of rivastigmine in acute scopolamine-induced amnesia and chronic colchicine induced cognitive dysfunction animal models, and validate the best formulation by employing pharmacokinetic and pharmacodynamic (PK-PD) modeling. Nasal liposomal rivastigmine formulation showed the best pharmacokinetic features with rapid onset of action (Tmax = 5 min), higher Cmax (1489.5 ± 620.71), enhanced systemic bioavailability (*F* = 118.65 ± 23.54; AUC = 35,921.75 ± 9559.46), increased half-life (30.92 ± 8.38 min), and reduced clearance rate (Kel (1/min) = 0.0224 ± 0.006) compared to oral rivastigmine (Tmax = 15 min; Cmax = 56.29 ± 27.05; *F* = 4.39 ± 1.82; AUC = 1663.79 ± 813.54; t1/2 = 13.48 ± 5.79; Kel (1/min) = 0.0514 ± 0.023). Further, the liposomal formulation significantly rescued the memory deficit induced by scopolamine as well as colchicine superior to other formulations as assessed in Morris water maze and passive avoidance tasks. PK-PD modeling demonstrated a strong correlation between the pharmacokinetic parameters and acetylcholinesterase inhibition of liposomal formulation.

**Supplementary Information:**

The online version contains supplementary material available at 10.1007/s00210-021-02096-0.

## Introduction

With the increasing aging population and progressive nature of the disease, Alzheimer’s disease (AD) poses to be an oncoming epidemic with limited therapeutic strategies (Apostolova 2016; Feigin 2019). AD is the commonest cause of dementia, and dementia constitutes the fifth leading cause of death globally (2020 Alzheimer’s disease facts and figures 2020). It is characterized by the deposition of extracellular amyloid β plaques and intracellular tau neurofibrillary tangles, which leads to neuroinflammation, synaptic dysfunction, and, subsequently, progressive neurodegeneration. Lately, the drug pipeline for AD remains dry with failed clinical trials and no new FDA approved drugs since 2003. The existing few therapeutic drugs, rivastigmine and donepezil, can only alleviate the symptoms (Sharma 2019; Deture and Dickson 2019).

Degeneration of cholinergic neurons and the resulting loss in cholinergic transmission promotes the cognitive decline in AD patients. Cholinesterase inhibitors represent the significant fraction of currently available drugs as they primarily enhance the cholinergic neurotransmission in the brain by delaying the degradation of acetylcholine available in the synaptic clefts (Sharma 2019). Donepezil, galantamine, and rivastigmine are the FDA-approved cholinesterase inhibitors recommended for treatment of AD. Rivastigmine is a second-generation carbamate derivative and reversible, non-competitive cholinesterase inhibitor widely used in mild to moderate AD cases, and studies propose maximal therapeutic benefits with early and continuous treatment. It has salient features that it inhibits both acetyl and butyryl cholinesterase, with more selectivity to brain AchE, and without hepatic metabolism by the CYP-450 system leading to lesser drug interactions. The recommended dose for improving the cognitive symptoms is 6–12 mg/day, b.i.d, p.o. Despite its advantages, it has poor BBB penetration with log P predicted as 2.45 and has low bioavailability of 36–40% due to its hydrophilic nature as well as the extensive first-pass metabolism leading to a short plasma elimination half-life (1.5 h) (Jann 2000). Rivastigmine oral therapy exhibits high incidence of systemic adverse effects, particularly gastro-intestinal, multiple dose requirements, higher intra-individual variability, and unpredictable systemic exposure in comparison to other cholinesterase inhibitors (Khoury et al. 2018). A lately developed transdermal patch of rivastigmine has overcome some of its shortcomings by improving the tolerability; however, brain penetration is still poorer (Dhillon 2011). Therefore, alternative brain targeted delivery strategies for rivastigmine are the need of the hour.

Intranasal drug delivery, a non-invasive approach, has gained immense interest recently because of its direct transport of drugs to the brain through olfactory and trigeminal nerve pathways, circumventing the brain barriers. Further, this route provides rapid onset of action besides avoiding the gastrointestinal breakdown and first-pass metabolism (Agrawal 2018). Thus, the nasal route can be exploited in the present study to deliver rivastigmine efficiently to the brain. Despite the direct access to the brain, limited drug absorption and nasal permeability remain a challenge with nasal delivery of hydrophilic drugs. To overcome this, nanocarriers such as PLGA nanoparticles and liposomes represent efficient vehicle systems to deliver the hydrophilic cargo (rivastigmine) to the brain (Vieira and Gamarra 2016). Therefore, the present study aims to evaluate the pharmacokinetic and pharmacodynamic profile of liposomal and nanoparticle formulations of rivastigmine delivered nasally to treat dementia.

## Materials and methods

### Animals

**Fig. 1:**
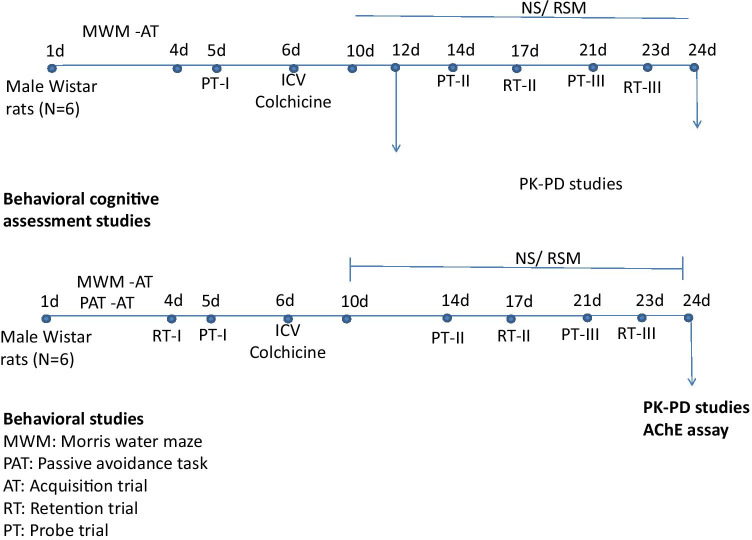
Experimental protocol for acute and chronic models of dementia followed in the study

The study was performed on healthy male Wistar rats aged 10 months, weighing 275 ± 10 g procured from the central animal house research facility, Manipal University, Manipal. Previous study from our laboratory showed a significant impact of gender on the pharmacokinetics of rivastigmine. Female rats showed significantly higher plasma drug levels and t1/2, besides lower clearance rate. On the other hand, male rats exhibited a 2.5-fold greater elimination rate constant than female rats. Based on this, males were chosen for the study (Arumugam 2009). All experiments were performed as per the guidelines of the Institutional Animal Ethical Committee of Manipal University. Rats were housed in polyacrylic cages in the animal house at controlled conditions temperature 22 ± 2 °C, humidity 60 ± 2%, and 12-h diurnal light cycle. Water and food in the form of dry pellets were provided ad libitum. All behavioral experiments were carried out from 18 to 21 h of the day in a room adjacent to that in which the rats were housed. The study design is depicted in the Fig. 1.

### Chemicals

Acetylthiocholine iodide (ATCI), scopolamine hydrobromide, and colchicine were purchased from Sigma Chemicals Co., USA. Rivastigmine hydrogen tartrate (99%) was obtained as a gift sample from Dr. Reddy’s Laboratories Limited, Hyderabad, India. Venlafaxine (internal standard) was kindly donated by Torrent Pharmaceuticals Limited, Ahmedabad, India. HPLC grade acetonitrile, methanol, and analytical grade ammonium acetate were obtained from Merck Chemicals, Mumbai, India. All other chemicals used were of analytical grade.

### Preparation of liposomes

Rivastigmine liposomal suspension was prepared by the lipid layer hydration method. Briefly, solution of soya lecithin and cholesterol (4:1) solubilized in chloroform was transferred to round-bottom flask. Flask was fixed to a rotary vacuum evaporator immersed in a thermostat water bath set at 45 °C and rotated until a dry lipid film was deposited on the flask walls. The aqueous phase (rivastigmine in PBS, pH = 7.4) was added and vortexed until the entire film deposited was consumed. The suspension was exposed to 3 freeze–thaw cycles, and the final preparation was stored at 4 °C. These liposomes were evaluated by optical microscope as well as scanning electron microscope (SEM). Drug content analysis was carried using a UV spectrophotometer. The formulation was characterized and in vitro release studies were carried (Arumugam 2008).

### Formulation development and characterization of PLGA nanoparticles

Rivastigmine-loaded nanoparticles were prepared by nanoprecipitation method. Dispersed phase consisting of 100 mg of PLGA (RG 503H, 50:50, 34 kDa) was dissolved in 2 mL of acetone and mixed well. The dispersed medium comprised 10 mL of PVA (1 w/v %, pH 8.5), poloxamer 188 (0.5% w/v), and 10 mg of rivastigmine and mixed thoroughly. The organic phase was added drop wise (1 mL/min) into the aqueous phase and stirred at 300 rpm in a magnetic stirrer. Following evaporation of acetone by continuous stirring for about 6 h at room temperature, then, nanoparticles were purified. Nanosuspension was ultracentrifuged at 20,000 rpm at − 10 °C for 1 h, and supernatant was discarded, and pellet was resuspended in 3 mL of mannitol (10% w/v) solution. The resulting nanosuspension was frozen for 12 h at − 70 °C and then lyophilized at − 40 °C/50 m Bar for about 48 h. The freeze-dried, moisture-free nanoparticles were stored in a type I glass container and kept at 4 °C until further analysis.

The developed formulation was characterized further by in vitro techniques to understand their properties like zeta potential, in vitro release, long-term stability, and surface morphology, and the results are produced in Table 1.

**Table 1:** In vitro characterization of rivastigmine PLGA nanoparticles

Parameters	Description
Appearance	White, free flowing powder
pH (0.1% w/v)	5.11 ± 0.20
Particle size Z-average (nm)	343.25 ± 50.55
Zeta potential (mV)	− 21.00 ± 4.58
Polydispersity index	0.28 ± 0.14
Entrapment efficiency (%)	14.82 ± 4.1
Loading efficiency (%)	0.82 ± 0.24
Surface morphology	Smooth surface and spherical
Physical state	Amorphous
Release kinetics/mechanism	Higuchi/non- Fickian
Stability	Stable
Reconstitution	Complete suspension

#### Entrapment and loading efficiency

Encapsulation efficiency (EE) of rivastigmine nanoparticles was carried out by ultracentrifugation method. Known amount of freeze-dried rivastigmine-loaded PLGA nanoparticles were weighed and dissolved in acetone. Ten milliliter of suspension was taken in the centrifuge tube, crimped, and then ultracentrifuged (WX Ultra 90, Thermo scientific USA) at 41,900 rpm for 1 h at 4 °C. The supernatant having free drug was collected cautiously, and pellet was reconstituted with PBS solution (pH 7.4). Both supernatant and pellet were analyzed by the HPLC method. The drug EE and loading efficiency (LE) was calculated by the following equations: (Li 2009)

$$\mathrm{\% EE}=(\mathrm{Amount of rivastigmine present in the nanoparticles})/(\mathrm{Initial drug amount})\times 100$$

$$\mathrm{\% LE }=(\mathrm{Amount of rivastigmine present in the nanoparticles})/(\mathrm{Total weight of nanoparticles})\times 100$$

#### Reconstitution test

Lyophilized rivastigmine-loaded nanoparticles were suspended in water/saline (0.1% w/v), and solution was vortexed for 5 min. Resulting solution was observed for any sedimentation or precipitation for up to 1 h at room temperature and checked for their reconstitution efficiency.

#### Particle size, polydispersity index (PDI), and zeta potential

Particle size, polydispersity index, and zeta potential of nanoparticles were measured with a dynamic light scattering method (Zetasizer nanoZS, Malvern instruments, UK) with an He–Ne laser beam at a wavelength of 633 nm at 25 °C (scattering angle of 90°). The concentration of nanoparticles used was 0.1% w/v in Milli-Q water. Sample was filled (3/4) in disposable cuvette, and then, average particle size distribution and PDI were determined. Zeta potential measured using clear disposable zeta cell and all measurements were carried out in triplicate at 25 ± 1 °C.

#### Physical appearance and pH measurement

In order to maintain the isotonicity of the resuspended nanoparticles (in water/saline), pH was determined using pH meter (Systronics pH meter, Bangalore). Physical appearances of rivastigmine encapsulated freeze -dried nanoparticles were observed.

#### Surface morphology

The surface morphology of the freeze-dried optimized rivastigmine nanoparticles were studied for their shape, surface microstructure (porous/hollow/aggregated mass and individual particle sizes were determined using SEM (Leo Corporation, Zeiss leica, UK), and TEM (Morgagni 268, Olympus).

##### Scanning electron microscopy (SEM)

The surface morphology of liposomes was studied using scanning electron microscopy. The resolution of 3.5 nm was used with secondary electron image display. The liposomes were coated with gold–palladium alloy (150–250 Å) using a sputter coater. The coater was operated at 2.2 kV, 20 mV, 0.1 torr (argon) for 90 s. An accelerating voltage of 15 kV was used. The magnification of × 200 was used to scan the liposomes. The instrument was operated at 60–80 K; Leo 435 VP variable pressure scanning electron microscope manufactured by Leo Corporation, Zeiss leica, UK, was used (Fang et al. 2006).

##### Transmission electron microscopy (TEM)

Fifty microliter of nanoparticles dropped in the parafilm and copper grid were kept above the samples and waited for 1 min. Then, the copper grid was taken out and dropped in the 2% phosphotungstic acid, and waited for 30 s. Afterwards, copper grid was taken out and dried with help of tissue paper, and then placed under the TEM microscope (Morgagni 268, Olympus). Pictures were taken at various magnifications (× 13,000), and accelerating voltage 70 kV and data were analyzed using Olympus soft imaging viewer.

#### In vitro release studies and its release kinetics and mechanism

In vitro release studies were performed using dialysis sac (Sigma membrane, MW cut-off 13 kDa) method. Two milligram equivalent of rivastigmine-loaded nanoparticles were weighed and suspended in 2 mL of phosphate buffer saline (pH 7.4). Samples were withdrawn at predetermined time intervals and analyzed by HPLC method. In order to maintain sink conditions, an equal volume of PBS was replaced. The whole experiment was performed at 250 °C. Based on the cumulative drug release vs. time profiles, the release kinetics models and mechanism were proposed (Akbuǧa and Bergişadi 1997).

#### Stability studies

The formulation of rivastigmine nanoparticles were subjected to accelerated (25 ± 2 °C, 60 ± 5%) and long-term stability conditions (2–8 °C) using stability chambers (Thermo stability chambers, UK) for a period of 6 months. The freeze-dried nanoparticles were stored in a glass container (type I) and sealed with a crimper after flushing with nitrogen. Samples were withdrawn at 15, 30, 60, 90, and 180 days, and the drug content, particle size, polydispersity index, and zeta potential were determined.

### Pharmacokinetic study

Four separate groups of rats (*N* = 4), INRSM (intranasal rivastigmine solution), INRL (intranasal rivastigmine liposomes), INRNP (intranasal rivastigmine nanoparticles), and ORSM (oral rivastigmine solution) were used for pharmacokinetic studies. The overnight fasted rats were administered with different rivastigmine formulations 30 min following scopolamine treatment. 0.2 mL blood was collected from the retro-orbital vein in Eppendorf tubes containing disodium EDTA (10% w/v) at 0, 5, 10, 15, 20, 30, 45, 60, 90, 120, 180, and 240 min after rivastigmine. Samples were then centrifuged at 10,000 rpm for 5 min, and plasma was stored at − 70 °C until analysis. The plasma samples were used to estimate rivastigmine concentrations and for the AChE inhibition assay.

After a 15-min interval, rats were sacrificed and brains were isolated. The whole brain was rapidly weighed and homogenized in PBS (10%w/v), and 500 µL of this whole homogenate was taken in a 1.5-mL centrifuge tube, centrifuged at 10,000 rpm for 5 min at − 10 °C, and clear supernatant 100 µL was taken for the AChE activity.

Similarly, rats treated with colchicine ICV were treated with different rivastigmine formulations and sampled at different time intervals until 2 h on the 12th day post-colchicine administration. On day 24 after the induction of dementia, rat brain homogenates were prepared as described above for AchE activity.

#### Extraction of rivastigmine

Extraction of rivastigmine from rat plasma was carried out using the liquid–liquid extraction (LLE) technique. Briefly, to an aliquot of 100 µL of rat plasma, 10 µL of IS (1 µg/mL) was added and vortexed for 30 s. Then, 1 mL of TBME tertiary methyl butyl ether) was added and vortexed for 10 min, thereafter centrifuged at 10,000 rpm for 5 min. From the supernatant, 0.9 mL of organic solvent was aspirated and evaporated in a turbo evaporator (Zymark, Hopkinton, MA, USA) at 50 ± 2 °C under the stream of nitrogen. The dried residue was reconstituted with 150 µL of mobile phase, and 50 µL was injected for the HPLC analysis. A full validation according to the US-FDA guidelines was performed in rat plasma. Plasma samples were analyzed by previously validated HPLC with fluorescence detection method (Arumugam 2011).

#### Rivastigmine quantification by HPLC (Arumugam 2011)

HPLC analysis was carried out on Shimadzu LC-20AD Prominence (Shimadzu Corporation, Kyoto, Japan) equipped with LC-20AD pump, RF-10A-XL fluorescence detector, column oven (CTO-10AS VP), autosampler SIL-20AC HT, and LC solution version 1.24 SP1. The column oven temperature was maintained at 25 °C. The chromatographic separation was achieved by Intersil, ODS-3 V, C18 (250 × 4.6 mm, 5 µm) column (GL Sciences Inc., Japan). Isocratic elution was performed with ammonium acetate buffer (20 mM, pH 4.5) and acetonitrile 74:26 (v/v) as mobile phase. The flow rate was maintained at 1 mL/min, and the injection volume was 50 µL. Fluorimetric detection was used, and excitation and emission wavelength were 220 nm and 293 nm, respectively. The sensitivity of the detector was “medium”, and the response kept at 3. Venlafaxine (IS) was prepared separately in methanol to yield primary standard solutions with a concentration of 1 mg/mL.

#### Pharmacokinetic analysis

The pharmacokinetic parameters were calculated using non-compartmental analysis, PK solution 2.1software (Summit Research Services, Montrose, Colorado, USA). The area under the curve (AUC) from zero to infinity is calculated using the trapezoidal method. The terminal elimination rate constant (β) was obtained by plotting log-linear concentration versus time; the corresponding half-life (t1/2β) was calculated by 0.693/β. Plasma drug concentration of rivastigmine at time zero (C0) was obtained by extrapolation of time-plasma concentration profiles. The clearance (CL) and volume of distribution (Vd) were calculated as follows:

$$\mathrm{CL }=\mathrm{Dose}/\mathrm{AUC}0-\infty ,\mathrm{ Vd }(\mathrm{apparent})=\mathrm{ CL}/\upbeta$$

### Pharmacodynamic studies

The efficacy of the different formulations was assessed in an acute, scopolamine-induced amnesia model and a chronic model using colchicine-induced neurodegeneration.

In the acute study, the following groups were used (Table 2).

**Table 2:** Groups used in scopolamine-induced amnesia (acute model) in rats

S.No	Group (*n* = 6)	Model induction	Treatment
I	NC	Normal saline	Normal saline
II	SCP	Scopolamine 1 mg/kg (i.p) induced amnesia (acute model)	Normal saline
III	SCP + ORSM		Rivastigmine (2.5 mg/kg, p.o.)
IV	SCP + INRSM		Rivastigmine (2.0 mg/kg, IN)
V	SCP + INRL		Rivastigmine liposomal formulation (2.0 mg/kg, IN)
VI	SCP + INRNP		Rivastigmine nanoparticle formulation (2.0 mg/kg, IN)

*NC* normal control, *SCP* scopolamine, *ORSM* oral pure rivastigmine, *INRSM* intranasal pure rivastigmine, *INRL* intranasal liposomal formulation of rivastigmine, *INRNP* intranasal nanoparticle formulation of rivastigmine

In the chronic study, the following groups are used (Table 3).

**Table 3:** Groups used in ICV colchicine-induced dementia (chronic model) in rats

S.No	Group (*n* = 8)	Model induction	Treatment
I	SC	-	Normal saline
II	ACSF	ACSF only	Normal saline
III	COL	Colchicine 15 µg (ICV) induced dementia (chronic model)	Normal saline
IV	COL + ORSM		Rivastigmine (2.5 mg/kg, p.o.)
V	COL + INRSM		Rivastigmine (2.0 mg/kg, IN)
VI	COL + INRL		Rivastigmine liposomes (2.0 mg/kg, IN)

*SC* sham control, *ACSF* artificial cerebrospinal fluid, *COL* colchicine, *ORSM* oral pure rivastigmine, *INRSM* intranasal pure rivastigmine, *INRL* intranasal liposomal formulation of rivastigmine

### Experimental design

Scopolamine, rivastigmine, and other formulations of rivastigmine were prepared in normal saline freshly before administration. Rivastigmine solution was maintained at isotonic pH of 6.10 ± 0.20 to maintain nasal cavity pH (5.5–6.5). During the experiments, care was taken to maintain the normal functions of the nasal cavity by minimizing disturbance of the mucosa with mechanical manipulation. Rats were anesthetized with diethyl ether, and rivastigmine solution was administered through the rat nasal cavity. The nasal doses were given unilaterally to the right nostril (50 μL) of the rats using microtips (100 μL) attached to a micropipette. The rats were kept in the supine position for 2 min after administration of the dose. All experiments were carried out at 20 ± 5 °C (Krishnan 2017).

In the acute model, the rats were trained for Morris water maze (MWM) and passive avoidance test (PAT) tasks in the first 4 days. On the 5th day, 30 min after scopolamine administration, respective treatments were given to all the groups. Fifteen minutes thereafter, the test trials were conducted in MWM and PAT.

In the chronic model, the rats were trained for MWM and PAT tasks for the first 4 days followed by intracerebroventricular (i.c.v.) administration of colchicine on day 6. After establishing dementia on day 10 of the colchicine administration, the drug treatment was initiated from day 11 to 24. The effects of different drug treatments on dementia were assessed on days 14 and 21. On these particular days, the swim test was performed 15 min after the administration of different treatments and escape latencies; residence time and peripheral swim time were recorded (Nampoothiri 2017). The retention trials for PAT on day 17 and 23 were performed on days 17 and 23, 15 min after the administration of the drug. Following rivastigmine administration, 15-min lag time for assessment was chosen based on pilot PK and PD studies where Cmax was observed.

### ICV colchicine-induced dementia

On the 6th day, surgery was performed as described (Bokare et al. 2018). Rat was anesthetized with sodium pentobarbital (45 mg/kg, i.p.) and positioned in stereotaxic apparatus. The head was positioned in a frame, and a midline sagittal incision was made in the scalp. Two holes were drilled through the skull at coordinates (0.8 mm posterior to bregma, 1.8 mm lateral to the sagittal suture, and 3.6 mm beneath the cortical surface) to place the injection cannula. Rats were intracerebroventricularly infused with either artificial cerebrospinal fluid (ACSF; in mM: 147 NaCl, 2.9 KCl, 1.6 MgCl_2_, 1.7 CaCl_2_, and 2.2 dextrose) or 15 µg of colchicine in 5 µL ACSF using a Hamilton microsyringe positioned in the injection cannula. To promote back diffusion, the microsyringe was left in place for 2 min following injection. The scalp was then closed with a suture. After surgery, all animals received gentamicin (5 mg/kg, i.p.) to prevent sepsis. Special care was taken during the postoperative period to provide food and water inside the cage of the rat. In sham-operated rats, surgery was identical except for injection of the solution.

### Behavioral assessment

#### Locomotor activity (Kaur et al. 2015)

Locomotor activity was assessed in animals using a digital photoactometer (INCO, India) which contains a cage that is 30-cm long and 30-cm deep with a wire mesh at the bottom. The apparatus was placed in a dark, light, and sound attenuated testing room. Before the locomotor task, animals were placed individually in the activity meter for 3 min for habituation. Then, the ambulatory movements were recorded for 5 min and expressed in terms of total photo beam counts for 5 min per animal.

#### Assessment of cognitive performance

Spatial learning and memory were assessed in the Morris water maze. Learning and memory were assessed in passive avoidance.

##### A. Morris water maze test 

A modified Morris water maze test was used (Nampoothiri 2017). The pool was positioned in the middle of a dimly lit testing room with distant visual clues that could be used by rats for spatial orientation. The maze consisted of a circular water pool (140 × 45 cm) divided into four equally sized quadrants (NE, SE, SW, and NW) by two imaginary diagonal lines running across the center of the pool. The pool was filled with water maintained at a temperature of 23 ± 2 °C. In the NE quadrant (target quadrant) equidistant from the center and edge of the pool, a platform of 25-cm height and 10-cm diameter was invisibly placed submerged 1 cm below the water surface. Rats were gently placed in each quadrant facing the wall and trained to locate the hidden black platform, and the trial was terminated when the rat reached the platform or after 60 s. Accordingly, rats were subjected to one session of four trials per day for four consecutive days. All rats were left on the platform for 30 s and then removed and towel dried. Inter-trial interval was kept a constant 180 s throughout the study. In the probe trial, platform was removed, and rats were allowed to swim for a 60-s trial period. Data was collected in the form of escape latencies to find the platform; percent of time spent over the target quadrant.

##### B. Passive avoidance test 

The passive avoidance apparatus consists of a larger illuminated compartment and a smaller dark compartment. Each consists of a square box, with a floor grid of 50 × 50 cm and wooden walls of 35-cm height in larger compartment. Larger box was illuminated by 15 W Philips CFL lamp. A smaller dark compartment of 15 × 15 cm with an electrified floor is connected to a constant current stimulator. An opening 6 × 6 cm in between the two compartments was closed using a transparent plexiglass sliding door. The test procedure was carried out in three phases, exploration, learning, and retention (Praveenkumar 2019).

First day, the animal was placed in the center of the illuminated larger compartment facing away from the entrance to the small darker compartment and allowed to explore the apparatus, i.e., both compartments for 3 min. Total time spent in the larger and smaller compartments and the number of crossings (from larger to smaller compartments and vice versa; a measure of exploratory behavior) were noted. On the second day, animal was placed in the illuminated larger compartment facing away from the entrance to the darker compartment. Once the animal entered the darker compartment, the entrance to the larger compartment was closed, and three strong electric foot shocks (50 Hz, 0.5 mA, 2 s) with 10-s intervals were applied. On the third day, rats were placed in the illuminated larger compartment and allowed to explore both the compartments for 3 min. The parameters assessed are latency time to enter into darker compartment, time spent in the darker and brighter compartments, and the number of crossings between the compartments.

### AChE assay

AChE activity and its inhibition by rivastigmine in rat plasma and whole-brain homogenate were estimated by ex vivo method using Ellman’s method (Pohanka 2015). In this assay, thiol ester acetylthiocholine was used instead of the oxyesteracetylcholine, as suggested by Ellman. Thiocholine, which was produced due to the hydrolysis of acetylthiocholine (ACTI) by AChE, was estimated by making it react with 5, 5′-dithiobis-(2-nitrobenzoic acid) (DTNB). The chemical, 5-thio-2-nitrobenzoic acid (yellow color complex), which was released due to reaction was estimated using a UV spectrophotometer in the range of 400 to 420 nm with maximal absorption at 412 nm. One hundred microliter of blank rat plasma was taken in the polypropylene vials. To this, 2.8 mL of phosphate buffer (0.01 M, pH 8) and 100 µL of DTNB reagents were added and mixed well. Then, 20 µL of ACTI (26.01 mg/mL of Milli-Q water) was added to the contents of the cuvette to initiate the enzyme reaction. The reaction kinetics was monitored at different time intervals (0, 1, 2, 3, 4 min), and absorbance was noted. 2.8 mL buffer (0.01 M, pH 8.0) and 100 µL DTNB reagent served as blank. Concentrations in the range of 0.03–30 mM were prepared using methanol as a diluent. Previously AChE activity of the blank plasma sample without adding rivastigmine was measured and that response was taken as a control. Substrate concentration and reaction time were optimized such that sufficient concentration of ACTI is available to bind with the enzyme to initiate the reaction and also it should not saturate the enzyme. In this study, varying concentrations (100–1000 µM) of ACTI were prepared, and reaction kinetics were observed. In general, the maximum time should be either 2–4 min or even less, because the enzyme might undergo degradation. In the present study, up to 4 min of reaction kinetics (changing the optical density or absorbance) was measured. One unit of AChE activity is defined as the number of µmol of acetylthiocholine iodide hydrolyzed per minute. The specific activity of AChE is expressed in moles/min/mg of protein (Kaur et al. 2015):

$$\begin{array}{c}\mathrm{AChE Activity }=(\mathrm{abs}.\mathrm{ per min }\times 1000 \times \mathrm{volume of enzyme})/(13600 \times \mathrm{TRM})\\ \mathrm{AChE Activity }=\mathrm{ above value }\times 1000 (\mathrm{nanomolar}/\mathrm{min}/\mathrm{mL})\end{array}$$

where 13,600 = molar extinction coefficient of DTNB: 1000-molar conversion factor: (TRM) total reaction mixture 3 mL: volume of enzyme (0.1 mL).

### PK-PD study

PK and PD studies were carried in scopolamine-induced amnesia rats.

#### A. PD analysis

PD (AChE) parameters were calculated using WinNonlin 5.2 (Pharsight Corporation, USA) software. Various PD models (Simple Emax, Inhibitory Emax, and Sigmoid Emax) were attempted using the software. The PD models were optimized based on the correlation (observed, predicted values), weighted correlation, Akaike information criterion (AIC), and Schwarz Bayesian criterion (SBC). A measure of goodness of fit was arrived at based on maximum likelihood, on the comparison of several models for a given data set, and the model associated with the smallest AIC. The best PD model was selected that had smaller AIC, BIC values, and good correlation (> 0.99) function.

#### B. PK-PD modeling

PK-PD model was developed to describe the effect of plasma concentration of rivastigmine on the AChE inhibition following intranasal administration. A total of nine PK models were developed to fit the pharmacokinetic profiles of rivastigmine. The best PK model was chosen based on the PK parameters output results standard error, % CV, residual values, correlation coefficient (observed vs. predicted), weighted correlation, AIC, and SBC values. Five PD models were developed and checked their fitting performance in terms of standard error, % CV, residuals, and diagnostic plots.

### Data analysis

Data were statistically analyzed using Graph Pad Prism 5.0 software. Mean values were analyzed by one-way ANOVA followed by Tukey post-hoc test. All data were expressed as the mean ± SE. *p* < 0.05 was considered statistically significant at 95% confidence interval.

## Results

### In vitro characterization of rivastigmine nanoparticles

**Fig. 2:**
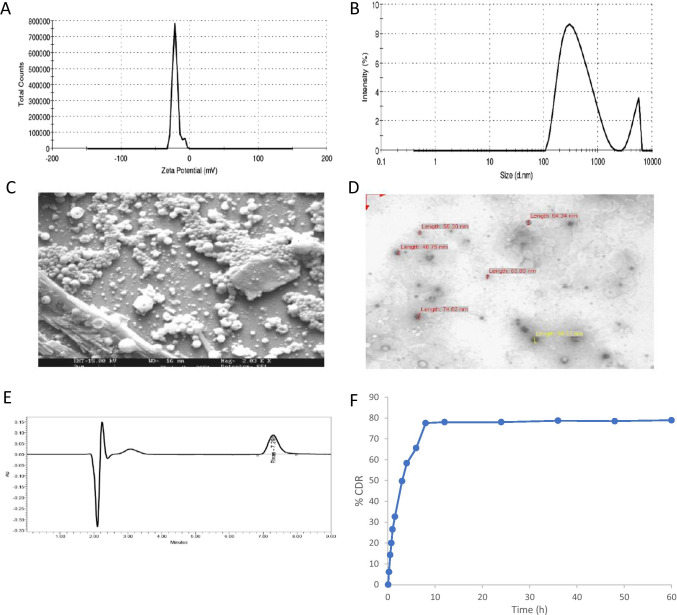
In vitro characterization of rivastigmine PLGA nanoparticles. (**a**) Zeta potential; (**b**) particle size measurements; (**c**) SEM and (**d**) TEM images of rivastigmine loaded nanoparticles; (**e**) typical HPLC chromatogram of rivastigmine in nanoparticles formulation; (**f**) in vitro release profile

Nanoprecipitation method formulated nanoparticles showed excellent reconstitution, quick suspension without any sedimentation. They exhibit smaller particle size with average particle size distribution of 343.25 ± 50.55 nM (Fig. 2b). This may be due to the less aggregation, rapid diffusion of miscible solvent into the stabilizer solution.

PLGA polymers show − 21.00 ± 4.58 mV of zeta potential (Fig. 2a). The negative surface charge is due to the presence of terminal carboxylic groups in the polymers and anionic nature of surfactants. High negative zeta potential indicates the electrostatic repulsion between particles, which would prevent the particle aggregation and could enhance the stability. No influence was exerted by rivastigmine on the zeta potential and electrophoretic mobility of PLGA nanoparticles.

Physical appearance of rivastigmine incorporated PLGA nanoparticles was found to be white color, free flowing, lower density, and powder form. Freeze-dried nanoparticles (0.1% w/v) were dissolved in water, and pH was found to be in the range of 4.67–7.37 at 25 °C, which indicates that nanosuspension is isotonic and there would not be a problem for nasal/parenteral route of administration of the developed formulation.

SEM analysis indicates a discrete, spherical shape, smooth, porous surface, and without aggregation (Fig. 2c). TEM analysis was carried out to study the surface morphology and particle size distribution of rivastigmine-loaded nanoparticles. Results show that PLGA nanoparticles were spherical shape and had narrow particle size distribution (Fig. 2d). The size of these nanoparticles (46–100 nm) was smaller than that determined by particle size analyzer (> 150 nm), presumably arising from the dry state measurement.

A biphasic release profile with an initial burst release up to 50%, within 2 h, 75% of drug within 6 h followed by sustained release of rivastigmine up to 60 h, whereas pure drug released 85% within 2 h (Fig. 2f). This may be attributed to adsorbed drug on the surface of nanoparticles which could have been released faster and then sustained or prolonged release from the polymer entrapped drug by matrix erosion/diffusion mechanism.

### Pharmacokinetic profiles of different formulation in scopolamine-induced amnesic rats

**Fig. 3:**
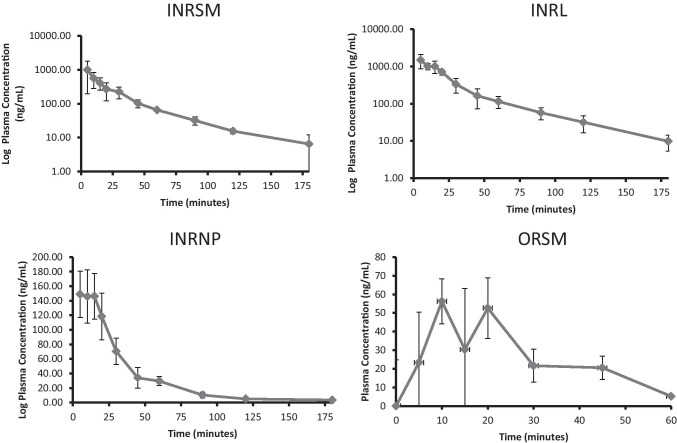
Plasma concentration–time curve of different formulation administered through intranasal or oral route in scopolamine induced amnesia rats (*n* = 4)

**Fig. 4:**
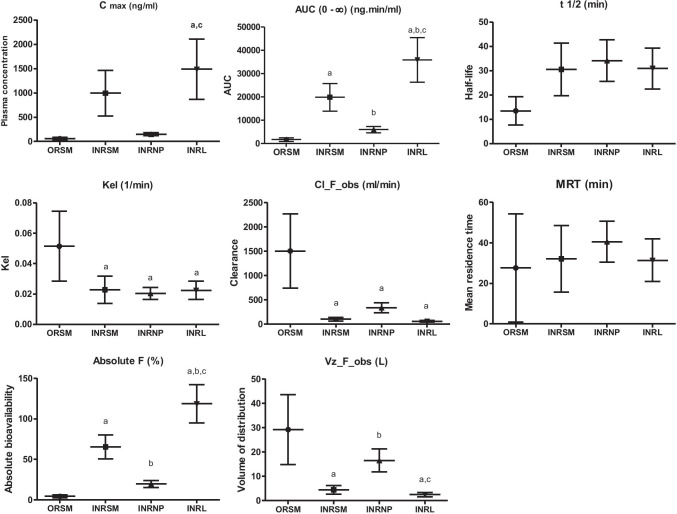
Pharmacokinetic features of different rivastigmine formulations. Maximum concentration (Cmax), area under the curve (AUC), elimination rate constant (Kel), half-life (*t* 1/2), clearance (Cl_F_obs), Mean residence time (MRT), absolute bioavailability (%F), volume of distribution (Vz_F_obs) of rivastigmine, and its formulations in scopolamine-induced rats. Data represented as mean ± SD, ANOVA followed by post-hoc test *a = *p* < 0.05 vs. ORSM; *b = *p* < 0.05 vs. INRSM; *c = *p* < 0.05 vs. INRNP

Nasally administered rivastigmine and its formulations (INRSM, INRL, INRNP) showed rapid absorption through nasal mucosa as demonstrated by high plasma drug concentrations at 5 min. The plasma drug levels declined rapidly in nasal rivastigmine nanoparticles (INRNP) and oral pure drug (ORSM) whereas maintained in INRSM and INRL (Fig. 3). The systemic availability (AUC) and peak plasma concentration were increased in the order of liposomes > nasal pure drug and PLGA nanoparticles. PLGA nanoparticles showed significantly lower systemic bioavailability and larger volume of distribution when compared to liposomes as well as nasal pure drug. Rivastigmine-loaded liposomes had a lower clearance rate compared to nanoparticles as well as nasal pure drug. The absolute bioavailability of rivastigmine was significantly higher for liposomes compared to pure drug and nanoparticles. Orally administered rivastigmine had the least Cmax, bioavailability (AUC), and maximum clearance where only a fraction of drug reached the systemic circulation. It had shown a larger volume of distribution (29.23 ± 14.35 L/kg) and an extremely higher clearance rate (1.50 ± 0.76 L/min) resulting in faster elimination from the plasma (Fig. 4).

### Effect of different rivastigmine formulations on scopolamine-induced memory deficit

#### A. Locomotor activity

**Fig. 5:**
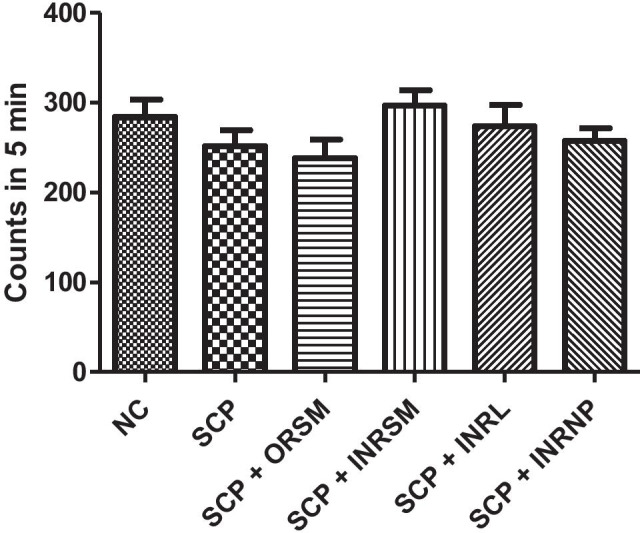
Influence of different formulations of rivastigmine on locomotor activity in scopolamine induced amnesia in rats

Locomotor activity as assessed by actophotometer is not significantly different among the groups as shown in Fig. 5.

#### B. Morris water maze 

**Fig. 6:**
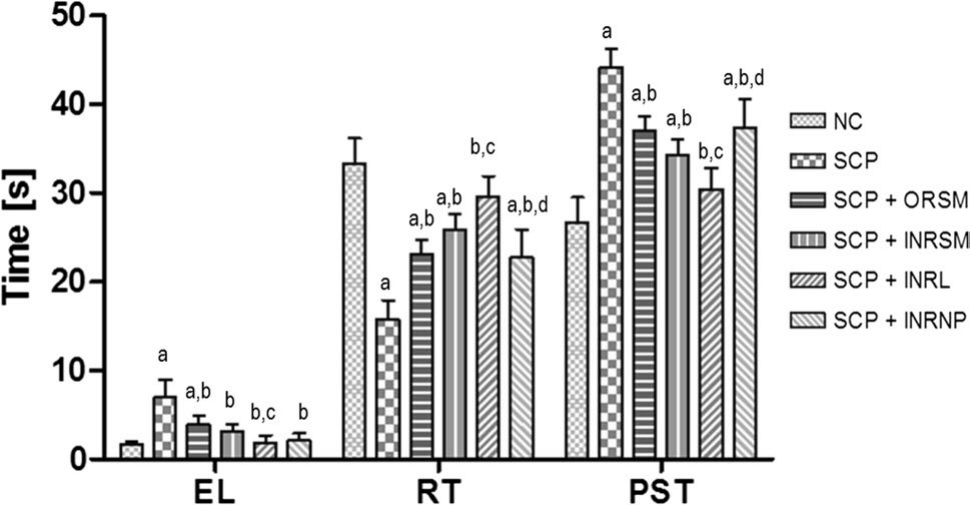
Influence of different formulations of rivastigmine in scopolamine induced amnesia on MWM task parameters: EL, escape latency; RT, residence time; and PST, peripheral swim time. Data represented as mean ± SD, ANOVA followed by post-hoc tests *a = *p* < 0.05 vs. NC; *b = *p* < 0.05 vs. SCP; *c = *p* < 0.05 vs. SCP + ORSM; *d = *p* < 0.05 vs. SCP + INRL

Time taken to find the platform is significantly higher in scopolamine-induced amnesic rats compared to control rats indicating the memory deficit in these rats (Fig. 6). Meanwhile, all rivastigmine-treated groups exhibited significantly shortened escape latency times, but the extent of reduction was highly significant in INRL group compared to the scopolamine-alone-treated group. The INRL group restored the memory almost comparable to normal control.

Scopolamine-alone-treated rats spent less time in the target quadrant and more time in the periphery compared to control as they failed to remember the platform location, indicating the loss of retentive memory in these rats. On the other hand, all the rivastigmine-treated groups spent significantly more time in the target quadrant and less time in the periphery indicating the restoration of retention memory in these rats. This beneficial effect is in the order INRL > INRSM > ORSM > INRNP (Fig. 6). Rivastigmine as a liposomal nasal formulation had shown better performance in working and reference memory in Morris water maze when compared to the other treatments in scopolamine-induced amnesia.

#### C. Passive avoidance test

**Fig. 7:**
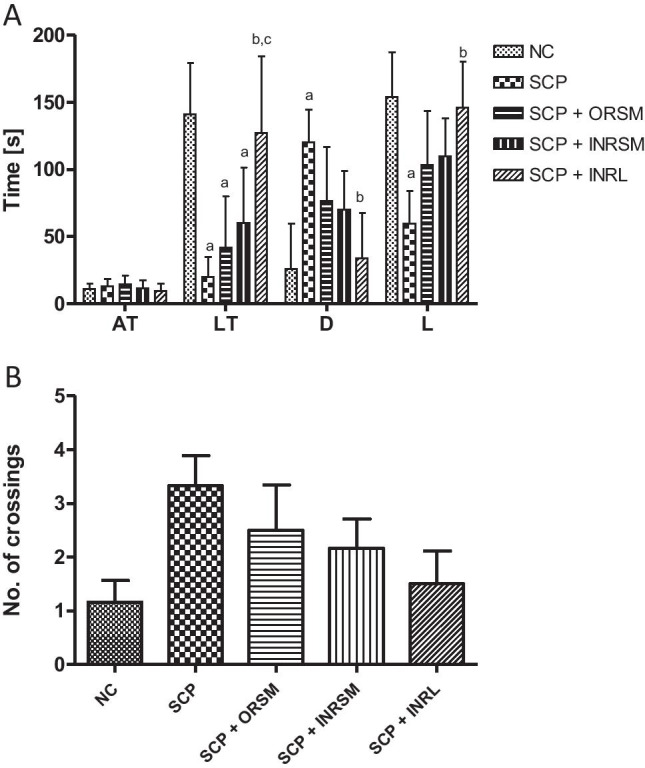
Effect of different rivastigmine formulations administered through different routes of administration in scopolamine induced amnesia on parameters of passive avoidance task. (A) AT (acquisition time), LT (latency time to enter into darker compartment), D (time spent in the darker compartment), L (time spent in the brighter compartment), and (B) number of crossings. Data represented as mean ± SD, ANOVA followed by post-hoc tests *a = *p* < 0.05 vs. normal control; *b = *p* < 0.05 vs. SCP; *c = *p* < 0.05 vs. SCP + ORSM

In the acquisition test trial, no significant difference was found among all the groups. Scopolamine-alone-treated rats have exhibited lower latency times, i.e., time taken to enter the darker compartment and spent higher time in darker chamber compared to control, as they fail to remember the electric shock in the darker compartment. Only the rivastigmine-loaded liposomal formulation given through the intranasal route has significantly reversed the effect of scopolamine, as evident by the enhanced latency times to enter the darker chamber and reduced residence time in the darker chamber as compared to scopolamine alone treated as well as ORSM groups, whereas all other treatment groups were not significant (Fig. 7A). There is no significant difference among the groups in the number of crossings between the darker and lighter compartments (Fig. 7B).

### Effect of different rivastigmine formulations on acetylcholinesterase activity in plasma and whole-brain homogenate in rats (ex vivo experiment)

**Fig. 8:**
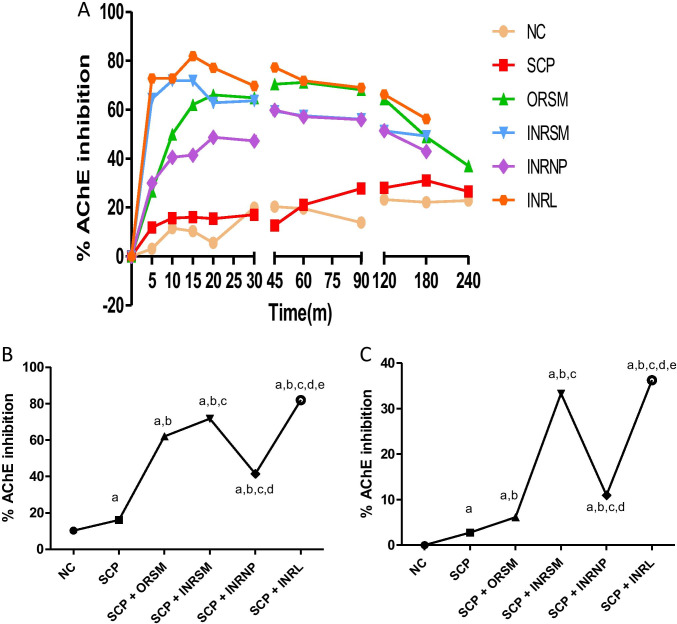
Influence of different rivastigmine formulations on % AChE inhibition in scopolamine induced amnesic rats. (A) At different time intervals with plasma samples. (B) At 15 min with plasma samples. (C) At 15 min with whole-brain homogenate. Data represented as mean ± SD, ANOVA followed by post-hoc tests. *a = *p* < 0.05 vs. normal control; *b = *p* < 0.05 vs. SCP; *c = *p* < 0.05 vs. SCP + ORSM; *d = *p* < 0.05 vs. SCP + INRNP; e = *p* < 0.05 vs. SCP + INRNP)

On comparison of all treatments, the peak AChE inhibition is noted at 15-min time interval by INRL (81.92%), followed by INRSM (71.95%) at 10 min, then ORSM (71.11%) at 60 min, and INRNP (59.86%) at 45 min in the plasma sample (Fig. 8a). At 15 min, INRL shows maximal AChE inhibition compared to all the treatment groups in plasma (Fig. 8b).

In the case of whole-brain homogenate, AChE inhibition was estimated only at 15-min interval. The peak AChE inhibition is noted by INRL (36.26%), followed by INRSM (33.34%), and then INRNP (10.97%) at last tailed by ORSM (6.16%) as shown in Fig. 8c.

### Effect of different rivastigmine formulations on colchicine-induced memory deficit

#### A. Locomotor activity 

**Fig. 9:**
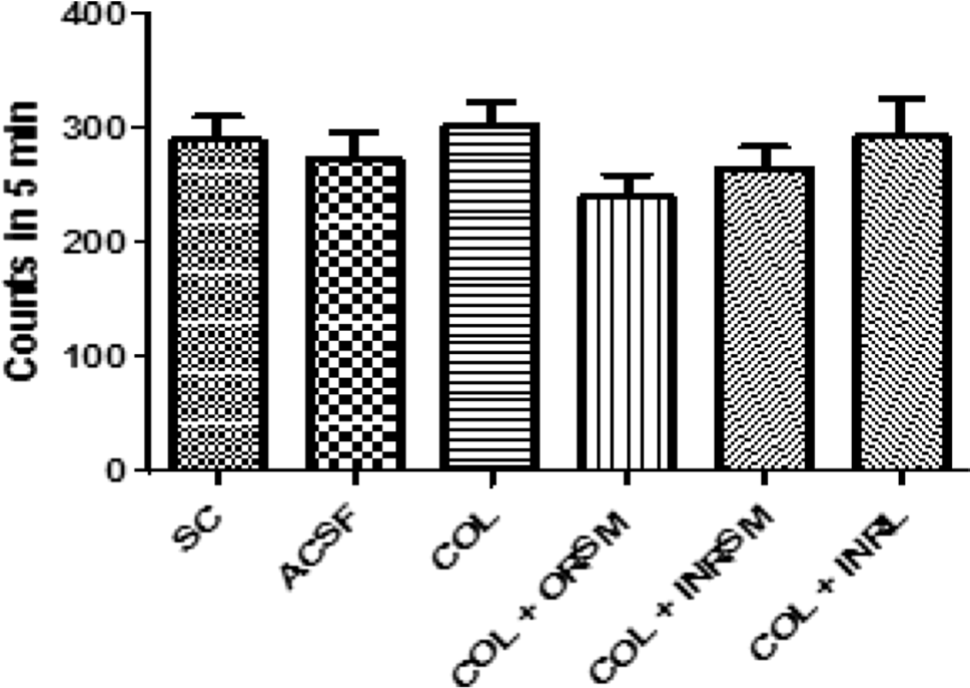
Influence of different rivastigmine formulations on locomotor activity in colchicine-induced dementia in rats

The spontaneous locomotor activity did not differ significantly between the groups as shown in Fig. 9.

#### B. Morris water maze 

**Fig. 10:**
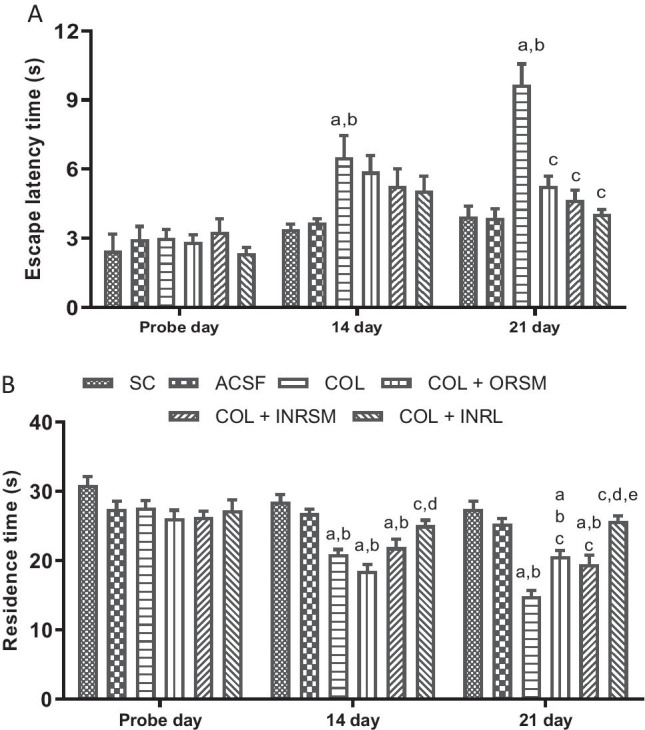
Influence of different rivastigmine formulations in colchicine induced dementia rats assessed by Morris water maze task parameters, (A) escape latency and (B) residence time. Data represented as mean ± SD, ANOVA followed by post-hoc tests. *a = *p* < 0.05 vs. SC; *b = *p* < 0.05 vs. ACSF; *c = *p* < 0.05 vs. COL; *d = *p* < 0.05 vs. COL + ORSM; *e = *p* < 0.05 vs. COL + INRSM

On the probe day, there was no significant difference in escape latency and residence times between the treatment groups. On day 14 (1st retention test) and day 21 (2nd retention test), the COL group displayed significantly increased escape latency and reduced time spent in the target quadrant over the SC and ACSF groups indicating the memory deficit induced in the colchicine-alone-treated rats. Meanwhile, all the treatment groups exhibited significantly reduced escape latency times, and increased time spent in the target quadrant over COL rats on the 21st day, and INRL was found to be significant over ORSM and INRSM groups in residence time (Fig. 10).

#### C. Passive avoidance test

**Fig. 11:**
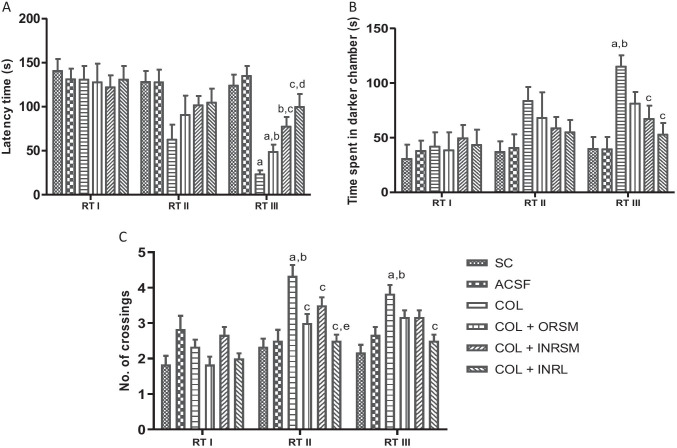
Influence of different rivastigmine formulations on (A) latency time to enter the dark chamber, (B) time spent in darker chamber, and (C) number of crossings on different retention test days during PAT in colchicine-induced dementia rats. Data represented as mean ± SD, ANOVA followed by post-hoc tests. *a = *p* < 0.05 vs. SC; *b = *p* < 0.05 vs. ACSF; *c = *p* < 0.05 vs. COL; *d = *p* < 0.05 vs. COL + ORSM; *e = *p* < 0.05 vs. COL + INRSM

No significant difference was found between the groups in all the parameters, latency to enter the darker chamber, time spent in the darker chamber, and the number of crossings assessed in the 1st retention test, which was performed before colchicine administration (Fig. 11). In the 2nd and 3rd retention test assessed on the 17th and 23rd-day post-colchicine administration, COL showed a significantly higher number of crossings and increase in time spent in dark chamber compared to sham and ACSF indicating the memory deficit induced by colchicine, as it failed to remember the electric shock. Meanwhile, all the treatment groups exhibited a reduced number of crossings as compared to COL in the 2nd retention test. In the 3rd retention test, INRL and INRSM significantly decrease time spent in dark chamber and number of crossings compared to COL (Fig. 11).

### Effect of rivastigmine formulations on acetylcholinesterase activity in plasma and whole-brain homogenate of rats in ICV colchicine-induced rats (ex vivo experiment)

**Fig. 12:**
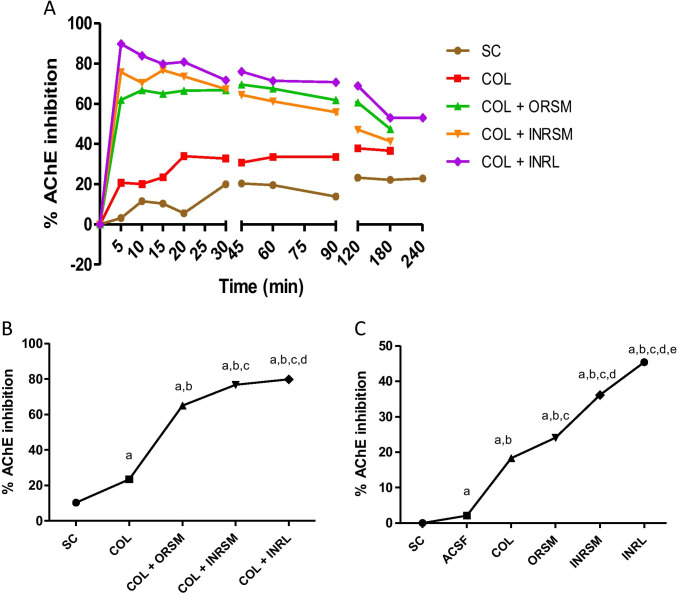
Influence of different rivastigmine formulations on % AChE inhibition in colchicine induced dementic rats. (A) At different time intervals with plasma samples. (B) At 15 min with plasma samples. (C) At 15 min with whole-brain homogenate. Data represented as mean ± SD, ANOVA followed by post-hoc tests (*a = *p* < 0.05 vs. SC; *b = *p* < 0.05 vs. ACSF; *c = *p* < 0.05 vs. COL;*d = *p* < 0.05 vs. COL + ORSM; *e = *p* < 0.05 vs. COL + INRSM)

Rivastigmine along with its formulations was tested for their effect on acetylcholinesterase inhibition in colchicine-induced dementia. On comparison of all treatments in rat plasma, the peak AChE inhibition was noted at 5-min time interval by INRL (89.86%), followed by INRSM (76.75%) at 15 min and ORSM (69.56%) at 45 min (Fig. 12A). At 15 min, AChE inhibition is significantly higher in INRL group compared to COL, ORSM, and INRSM plasma samples (Fig. 12B). The treatments with whole-brain homogenate are estimated at 15-min interval only, and the peak AChE inhibition is noted by INRL (45.38%), followed by INRSM (36.14%), and then by ORSM (24.09%) as shown in Fig. 12C.

### Pharmacokinetic and pharmacodynamic modeling in scopolamine-induced amnesia

#### PD model

The best PD model selected was Simple Emax ((Effect C = 0 at E0; Effect at C = infinity). This model was used to estimate the PD parameters namely Emax, EC50, E0, and AUE_0-t_ for plasma AChE ex vivo inhibition studies in rats. The PD effect of rivastigmine (% AChE inhibition) was in close agreement with the concentration–time course (i.e., PK profile) in plasma. Intranasally administered liposomes had significantly higher Emax (maximum inhibition 81.92%) than nanoparticles (59.86%) as well as pure drug (71.95%). Orally administered rivastigmine shows maximum AChE inhibition (71.11%) at 60 min (Fig. 8A).

#### PK model

Developed PK model was perfectly fitted into two-compartment models. Among the PK models developed, two-compartment, intravenous bolus, no lag time, microconstants, and first-order elimination seems to be the best fitted PK model to describe the PK parameters of rivastigmine-loaded liposomes. The developed model showed smaller % CV, AIC, and SBC values, and observed predicted concentrations were very close (smaller residuals), and correlation was more than 0.99. All these parameters indicate that rivastigmine PK parameters could be best fitted into the two-compartment model with first-order elimination as shown below equation: 
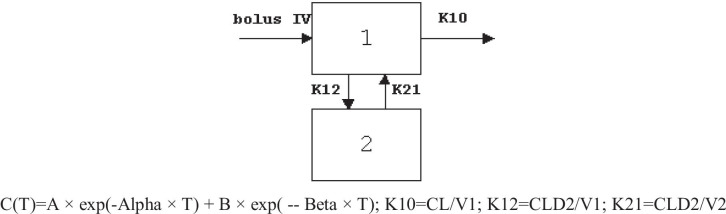


The inhibition of AChE in plasma could be best explained by a simple Emax model with Effect C = 0 at 0, C = infinity at Emax described by the equation as shown below equation:

$$\mathrm{E }=\mathrm{Emax }\times \mathrm{ Ce})/(\mathrm{Ce }+\mathrm{ ECe}50)$$

**Fig. 13:**
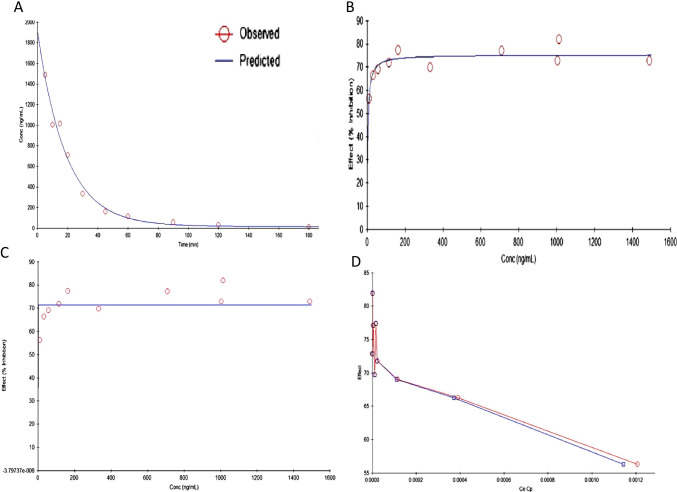
Predicted and observed models of rivastigmine-loaded liposomes following intranasal administration in scopolamine induced rats. (**a**) PK model predicted plasma concentration vs. time relationship. (**b**) PD model predicted plasma concentration vs. AChE inhibition. (**c**) PK-PD model predicted time vs. plasma AChE inhibition. (**d**) PK-PD model predicted hysteresis curve

The PK-PD results of liposomes are illustrated in Table 4. The developed PK model is found to best describe the disposition kinetics of rivastigmine-loaded liposomes following intranasal administration (Fig. 13a). PD data of rivastigmine in rat plasma is best fitted into the simple Emax model (Fig. 13b). Results reveal the reduction in AChE inhibition with time as the plasma concentrations decrease indicating the direct relationship between the PK and PD (Fig. 13c). The PK and PD of rivastigmine in plasma could be well described by these models that assume that no time delay between the activity and concentrations in plasma. Histersis curve indicates that drug concentration in plasma (Cp) and drug concentration in the effective site (Ce) are correlating as shown in Fig. 13d.

**Table 4:** Summary of estimated PK-PD parameters of rivastigmine-loaded liposomes in rat plasma

Parameter	Units	Std error	CV%
Emax	%	71.4033	3.46
ECe50	ng/mL	0.0000001	61,878.96
KEO		253,162.7668	25,778,658.00

The PK-PD link model showed a smaller standard error for KEO (rate of drug loss from the effective compartment) and other PD parameters. The predicted AChE inhibition or plasma concentration and experimental AChE inhibition or plasma concentrations are fairly matching. This result indicates that the PK-PD link model explains the time vs AChE inhibition very well.

## Discussion

Cholinesterase inhibitors are the best-established class of therapeutics for the treatment of dementia of Alzheimer’s type (DAT). Cholinesterase inhibitors enhance the availability of acetylcholine (ACh) at the synapse by inhibiting acetylcholine esterase enzyme inhibition (AChE inhibition). Acute and chronic treatment with cholinesterase inhibitors has been shown to improve cognitive function in animal models and patients with DAT (Sharma 2019). Rivastigmine, a reversible dual cholinesterase inhibitor, is a more tolerable and widely used choice of drug after donepezil among the available drugs. However, rivastigmine being hydrophilic and undergoing the first-pass metabolism exhibits low CNS bioavailability (Dighe 2019). Nanoformulations including liposomes and PLGA nanoparticles can encapsulate hydrophilic drugs and deliver efficiently nose to brain by improving their penetration, escaping the mucociliary clearance, and facilitating their transport across the mucosal barrier (Vieira and Gamarra 2016).

In the present study, intranasally administered rivastigmine liposomes had significantly improved pharmacokinetic parameters compared to the nasal pure rivastigmine, nasal rivastigmine nanoparticles, and oral rivastigmine. Liposomes produced rapid initial increase in plasma drug levels presumably due to the release of adsorped free drug present on the liposomal surface (Puri 2009). Moreover, liposomes had maintained the concentration up to 3–4 h and prevented the sudden peak to trough levels as in the ORSM group (Fig. 2). An ideal drug delivery system should have properties such as high Cmax, low clearance rate, and enhanced AUC, MRT, and half-life. The developed rivastigmine liposomal formulation shows such an increase in the Cmax and AUC (Fig. 4) indicating that it could reduce the toxic effects. These results are in accordance with the previous reports on the liposomal formulation for other drugs (Hong et al. 2019). A smaller volume of distribution and lower clearance rate was found for liposomes. This is possibly due to smaller particle size, hydrophilic nature of sterically stabilized liposomes which could have minimized the opsonization, and reduced the RES uptake leading to longer circulation time and higher MRT (Sercombe 2015). Liposomal formulation factors such as particle size, surface charge, chitosan coating, and PEGylation play a major role in the pharmacokinetic parameters of rivastigmine. On the other hand, rivastigmine-loaded PLGA nanoparticles showed significantly higher clearance rate, subsequently leading to a shorter half-life and less systemic exposure. The possible reason could be due to the higher uptake by RES/macrophages and the hydrophilic surface of nanoparticles (Behzadi 2017).

The cholinergic system in the basal forebrain plays an important role in learning and memory. Scopolamine interferes with memory and cognitive function by blocking M_1_ type muscarinic receptors in the brain regions (Nakayama and Sawada 2002). The present study compared the effects of a cholinesterase inhibitor, rivastigmine between pure form and the novel formulations administered via the intranasal route in the scopolamine-induced memory-impaired rats in the MWM and PAT tasks.

There was no significant difference among the treatment groups in locomotor activity assessed with an actophotometer indicating no effect of treatment on the motor activity of the animals. Rivastigmine at a dose of 2.5 mg/kg via the oral route inhibited the cholinesterase enzyme in the cortex and hippocampus by 20–30% and significantly reduced the effects of scopolamine on reference and working memory in MWM and PAT (Emerich and Walsh 1990). INRL treatment significantly reversed the memory deficit induced by scopolamine superior to other formulations as assessed in MWM and PAT tasks. Further, this was supported by pharmacokinetic parameters as well as acetylcholinesterase inhibition studies. The acetylcholinesterase inhibition was maximal for INRL in both rat plasma and whole-brain homogenate, and the order of inhibition was INRL > INRSM > ORSM and INRNP. Thus, the intranasal liposomal rivastigmine (INRL) treatment has a beneficial effect over the amnesia induced by scopolamine superior to the conventional oral treatment and also intranasal pure rivastigmine administration.

In the chronic model, colchicine binds irreversibly to tubulin to form tubulin dimers and prevents the addition of tubulin molecules to the fast-growing end, thereby inhibiting microtubule assembly and disrupting microtubule polymerization, resulting in cell death and subsequently cognitive impairment (Nakayama and Sawada 2002). In the present study, colchicine caused significant loss of memory as evident in MWM and PAT. It has been reported that colchicine produces time- and dose-dependent changes (anatomical, behavioral, and neurochemical) at a maximum of 14–21 days following the ICV colchicine administration (Emerich and Walsh 1990). As observed with the scopolamine model, the INRL group significantly rescued the memory deficit induced by colchicine administration. Thus, the present study demonstrated that rivastigmine along with its formulations was effective in enhancing the memory deficits in both the models employed. INRL was found to be more effective than the other treatments in all the parameters assessed in both models. The above statement was supported by acetylcholinesterase enzyme inhibition in plasma and whole-brain homogenates. The % inhibition was higher in the order of INRL > INRSM and ORSM. Here, the INRNP group, i.e., nanoparticle formulation, was excluded from the chronic model as it showed very poor results in the acute model (scopolamine-induced amnesia).

The study has some limitations; importantly, the pharmacokinetics of rivastigmine is different in males and females (Arumugam 2009), possibly due to the influence of sex hormones; and therefore, pharmacokinetic data from female rats is needed. Secondly, compromised BBB integrity during AD can also influence the drug concentrations in the brain. Therefore, pharmacokinetic study in animal model of AD with compromised BBB integrity, i.e., transgenic models, is needed. Also due to disease pathology and altered BBB, drug might get trapped in certain regions of brain. Therefore, estimation of the drug levels in different brain regions is recommended. Further, the nasal anatomy and physiology are different in rodents and humans. The olfactory region through which drug is supposed to be absorbed encompasses for 50% of nasal cavity in mice and rats, whereas in humans, only about 10% (Erdő et al. 2018). Hence, the pharmacokinetic data might change in humans. The nasal approach of drug delivery might be conveniently possible in early and moderate AD patients, and require the help of caretakers in late AD patients. Besides, the data on compliance of nasal drug delivery is lacking, which requires an immediate attention.

## Conclusion

Based on the results, nasal delivery of rivastigmine liposomes showed ideal pharmacokinetic characteristics with rapid absorption, enhanced systemic bioavailability, half-life, and mean residence time superior to other formulations. Besides, it reversed the memory deficit induced by acute (scopolamine) as well as chronic (colchicine) models and exhibited a good agreement between pharmacokinetic and pharmacodynamic activities. Therefore, the nasal route of rivastigmine liposomal formulation can be an ideal choice for treating dementia related to Alzheimer’s disease.

## Supplementary Information

Below is the link to the electronic supplementary material.

Supplementary file1 (XLSX 20 KB)

## Data Availability

Data is included in the form of an excel file.

## Abbreviations

PK-PD modeling: Pharmacokinetic and pharmacodynamic (PK-PD) modeling
PLGA: Poly D, L-lactic-co-glycolic acid
MWM: Morris water maze
